# Probable Danger from the Use of Cod-Liver Oil

**Published:** 1850-09

**Authors:** 


*Probable Danger from the use of Cod-Liver Oil.* By Dr. Benson.—At a recent meeting of the Surgical Society of Ireland, Dr. Benson made the following statement regarding cod-liver oil:

It was not to be expected that a remedy of so much power could be used with impunity in all cases. Having such efficacy in checking emaciation, in restoring the wasted flesh, and bringing back color to the faded cheek, it might be anticipated that it would in some cases of phthisis occasion a congested condition of the lung, and even give a tendency, or prove a predisposing cause to pneumonia; and this was, in fact, what he thought he had observed in some instances, and it was to this he begged to call the attention of the meeting.

It so happened that in almost every patient who died of phthisis under his care while using cod-liver oil, he found the lung congested and consolidated, not only in the neighborhood of the tubercles, but through nearly the entire of both lungs. This morbid condition, it is true, is often met with where no oil has been used, but he was struck with the greater frequency of it in the post-mortems he had made where the oil was freely administered. ('Dublin Med. Press.')—*Med. Gaz., Feb.* 1, 1850, *p.* 216.

